# Assessment of minimal active space CASSCF-SO methods for calculation of atomic Slater–Condon and spin–orbit coupling parameters in d- and f-block ions†

**DOI:** 10.1039/d1dt02346b

**Published:** 2021-10-01

**Authors:** Alvin J. Walisinghe, Nicholas F. Chilton

**Affiliations:** Department of Chemistry, School of Natural Sciences, The University of Manchester Oxford Road Manchester M13 9PL UK nicholas.chilton@manchester.ac.uk

## Abstract

Slater–Condon parameters and the spin–orbit (SO) coupling constants for various oxidation states of transition metal ions (3d/4d/5d) and trivalent f-block ions were calculated using minimal active space complete active space self-consistent field (CASSCF)-SO methods in OpenMolcas. The SO coupling constants have a quadratic relationship to atomic number *Z* for a fixed d^*n*^ configuration, as do those for the trivalent lanthanides where configuration also changes as a function of *Z*. Compared to experimentally-derived values, minimal active space CASSCF-SO approximates SO coupling constants within *ca.* 200 cm^−1^, which is usually <10% error for 4d^*n*^, 5d^*n*^ and 4f^*n*^ configurations, but up to 30% error for 3d^*n*^ configurations. Slater–Condon parameters are usually overestimated on the order of 10–50%, arising from a lack of dynamic correlation in the method, and thus we do not recommend minimal active space CASSCF-SO methods where accurate term excitation energies are required. However, the error in the Slater–Condon parameters appears to be systematic for divalent 4d and trivalent 4f ions such that scaling may be a useful approach where computational resources are limited, but this is not the case for 3d ions. Hence, caution is advised when using CASSCF-SO methods for comparisons with spectroscopic data, wherein only qualitative results can be expected, and methods accounting for dynamic correlation effects (such as CASPT2 or NEVPT2) should be employed if more quantitative results are required.

## Abstract 2

CASSCF-SO calculations in OpenMolcas are used to determine Slater-Condon and SOC parameters for 3d^*n*^, 4d^*n*^, 5d^*n*^ and 4f^*n*^ ions. We quantify the error of minimal active space approaches and compare to other similar methods.

## Graphical abstract

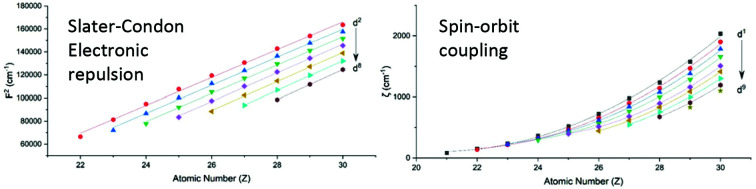

## Introduction

Computational chemistry has been an integral part of chemistry since the early 1950s, following the invention of digital computers in the 1940s. Computational chemistry methods were developed based on theoretical methods prevalent since the early 1900s. Nowadays, real chemical systems can mostly be solved to a very good degree of accuracy compared to experimental methods. However, there are still advantages in using simplified theoretical models as these often provide more clarity in explaining molecular properties or provide a flexible parameter-based framework in which to refine against experimental data, which is not possible with numerical-based solutions to many-body quantum problems.

Of the many theoretical methods for studying spectroscopic and magnetic properties in metal complexes, the methods of Condon and Shortley,^1^ and later recast in spherical tensor formalisms by Racah,^2^ have continued to find common application. In these methods, the quantum states are usually simplified to considering those of only the metal atom or ion valence electrons, where the angular parts of the atomic or ionic wavefunction are treated exactly and integrals over radial part of the wavefunction *R^n^_l_*(*r*) are treated as parameters (see eqn (1)). For instance, this allows one to consider the manifold of states arising from d^*n*^ or f^*n*^ configurations (and indeed including excited configurations such as f^*n*−1^d^1^, *etc*.) and transition moments between them, with the Hamiltonian equation (2) (here the first term describes the interelectronic Coulomb repulsion and the second term is the spin–orbit (SO) coupling). In such cases, the Coulomb-repulsion between electrons in these manifolds, which gives rise to the separations of ^2*S*+1^*L* electronic terms, are described by the Slater–Condon parameters *F*^*k*^ (eqn (3), which enter into the first term of Hamiltonian equation (2) by means of exact solution to the angular part of the operator^1^) for the single configuration approximation, which are sometimes expressed as Slater integrals *F*_*k*_ (eqn (3) and Table 1), or as Racah parameters for d^*n*^ configurations (eqn (5) and (6));^3^ the difference between Slater–Condon parameters *F*^*k*^ and Slater integrals *F*_*k*_ is merely one of convention, where the latter avoid occurrence of fractional coefficients arising from integrals of spherical harmonics.^1^ A single Slater–Codon parameter *F*^2^ is sufficient in describing a p^*n*^-system, whereas *F*^2^ and *F*^4^ are required to describe a d^*n*^-system and all three *F*^2^, *F*^4^ and *F*^6^ are required for f^*n*^-systems.^4^1
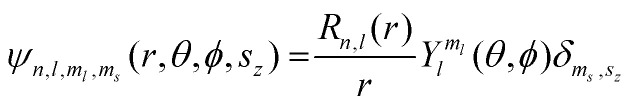
2
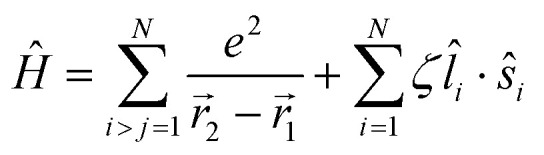
3
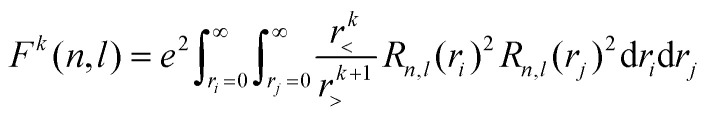

4 *F*^*k*^ = *F*_*k*_*D*_*k*_

5
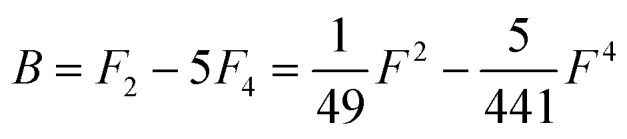
6
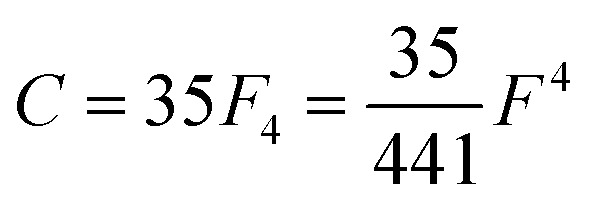


**Table 1:** D_*k*_ parameters for s, p and d orbitals

	*D*_2_	*D*_4_	*D*_6_
p	25		
d	49	441	
f	225	1089	7361.64

Moving beyond simulations of free atoms and ions, the environment is parameterised using the crystal field (CF) formalism, of which there are many varieties,^5^ and are of great utility in interpreting spectral data of complexes.^6,7^ While such model Hamiltonians do not account for metal–ligand covalency, polarisation, screening, *etc*., the parameters are amenable to optimisation compared to experimental observations (for instance *via* spectroscopy), and hence “experimental” wavefunctions can in principle be determined. These methods form a super-set encompassing spin Hamiltonian methods, such as those used to model molecular magnetic data^8^ and electron paramagnetic resonance spectroscopy.^9^ The utility of moving beyond spin Hamiltonians, which consider only low-lying electronic states, to explicit d^*n*^ or f^*n*^ configurations is that higher-order effects, such as deviations from the Landé interval rule^10^ and *J*-mixing,^11^ are implicitly included.^12^

Multi-configurational complete active space self-consistent field (CASSCF) methods, pioneered by Roos and co-workers,^13^ have seen an enormous rise in use recently for calculation of magnetic and spectroscopic properties of metal complexes, owing to the simple choice of active space of the d^*n*^ or f^*n*^ manifolds (the so-called “minimal active space”), and the availability of the single_aniso module^14,15^ in (Open)Molcas,^16^ and the *ab initio* ligand field theory (AILFT) module^17^ in Orca.^18^ The most-common implementations of CASSCF methods use scalar-relativistic Hamiltonians in a spin-free formalism,^19^ where a decoupling method is employed to transform the four-component Dirac Hamiltonian into a two-component method.^20^ This allows orbital optimisation with CASSCF in a spin-free formalism, where spin (and hence SO coupling) is introduced in a state-interaction picture after variational orbital optimisation.^21^ While such CASSCF calculations account for the static correlation of (near-)degenerate orbitals, often found in metal complexes which make single-determinant methods such as Hartree–Fock and density-functional theory inappropriate, and hence these calculations provide excellent qualitative results,^22^ CASSCF calculations lack the dynamic correlation of the exact configuration interaction (CI) solution.^23^ Dynamic correlation can, in principle, by introduced using perturbative methods such as CASPT2^24^ or NEVPT2,^25–27^ however these are extremely costly methods. Hence, it is commonplace to simply perform CASSCF calculations without such corrections.

Given the prominent use of minimal active space CASSCF-SO calculations with OpenMolcas for predicting electronic structure of coordination complexes, we decided to systematically explore the performance of this approach in predicting the electronic structure of metal ions. We employ minimal active space calculations for metal ions of varying oxidation states across the periodic table, accounting for all possible spin states in each d^*n*^ or f^*n*^ configuration, then recast the results into Slater–Condon and SO coupling parameters, and compare these to experimentally-determined parameters where possible, as well as results from the similar methods of AILFT and ligand field density-functional theory (LF-DFT). Compared to experiment, we find that minimal active space CASSCF-SO calculations generally overestimate the Slater–Condon parameters on the order of 20–50% (with some outliers in the 3d dataset which are underestimated by 1–20%), while SO parameters are overestimated by 5–30% for 3d ions, generally predicted within ±10% for 4d and 5d ions, and usually overestimated by 2–10% for trivalent 4f ions. Thus, we urge caution on the use of minimal active space CASSCF-SO calculations for the prediction of high-energy spectroscopic data and argue that dynamic correlation must be accounted for.

## Methods

We employed the OpenMolcas program^16,28^ to perform CASSCF-SO calculations on each of the metal ions herein. We used ANO-RCC-VQZP basis sets^29^ and atomic mean-field integrals (AMFI) to obtain the SO coupling Hamiltonian.^30^ We used the “ATOM” supersymmetry keyword to enforce spherical symmetry for the optimised atomic orbitals. State-averaged CASSCF (SA-CASSCF) optimisations with an *n* in 5 (d-block) or *n* in 7 (f-block) active space were performed for all possible roots for a given configuration and spin multiplicity (Tables 2 and 3); owing to computational limitations we have not explored Eu(iii), Gd(iii) and Tb(iii). Additionally, we have performed multi-state CASPT2 (MS-CASPT2)^31^ and extended multi-state CASPT2 (XMS-CASPT2)^32,33^ calculations for Co(ii) and Pr(iii), to illustrate the effects of corrections for dynamic correlation, using all roots from SA-CASSCF and an imaginary shift of 0.1. Following either SA-CASSCF or (X)MS-CASPT2, all roots were mixed with SO coupling. The resulting SO eigenstates were transformed into the Russell–Saunders basis with well-defined spin, orbital and total angular momenta as follows: (i) the matrix representation of *Ŝ*^2^ in the SO eigenbasis was diagonalised to obtain the SO → *Ŝ*^2^ unitary transformation 
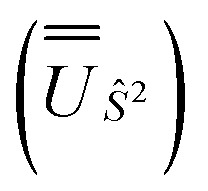
 and blocks of different *Ŝ*^2^ eigenstates were identified; (ii) the matrix representation of *L̂*^2^ in the SO eigenbasis was transformed into the *Ŝ*^2^ eigenbasis with 
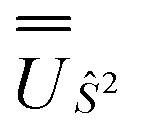
 and diagonalised within each eigenblock of *Ŝ*^2^, where the resulting eigenvectors were used to construct the SO → *Ŝ*^2^, *L̂*^2^ unitary transformation 
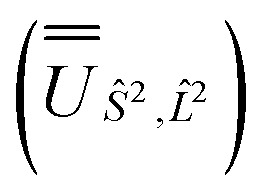
; (iii) the matrix representation of *Ĵ*^2^ in the SO eigenbasis was transformed into the *Ŝ*^2^, *L̂*^2^ eigenbasis with 
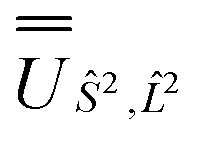
, and diagonalised within each eigenblock of *Ŝ*^2^, *L̂*^2^, where the resulting eigenvectors were used to construct the SO → *Ŝ*^2^, *L̂*^2^, *Ĵ*^2^ unitary transformation 
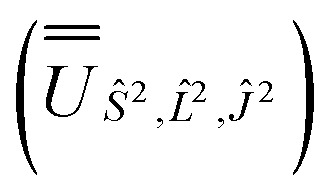
; (iv) the SO Hamiltonian of the electronic structure calculation (which is diagonal in the SO eigenbasis) was transformed into the *Ŝ*^2^, *L̂*^2^, *Ĵ*^2^ basis with 
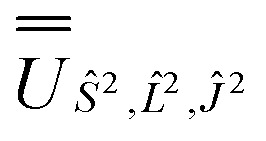
 and then diagonalised to return the original SO eigenstates and their composition in terms of the Russell–Saunders basis. Then, the Slater–Condon and SO parameters were first estimated from the average SO eigenvalues of each ^2*S*+1^*L*_*J*_ term, and subsequently refined by fitting the entire energy spectrum of the model Hamiltonian (eqn (2)) to the entire energy spectrum from CASSCF-SO. We note that this method is not restricted to minimal active space calculations and thus is broadly applicable beyond the scope of the present work. This code is under preparation and will soon be released as an open-source package.

**Table 2:** Spin multiplicities and roots included in the SA-CASSCF and SO coupling steps for d-block ions

d^*n*^	Spin multiplicity			Roots		
1	2			5		
2	3	1		10	15	
3	4	2		10	40	
4	5	3	1	5	45	50
5	6	4	2	1	24	75
6	5	3	1	5	45	50
7	4	2		10	40	
8	3	1		10	15	
9	2			5		
10	1			1		

**Table 3:** Spin multiplicities and roots included in the SA-CASSCF and SO coupling steps for f-block ions

f^*n*^	Spin multiplicity				Roots			
1	2				7			
2	3	1			21	28		
3	4	2			35	112		
4	5	3	1		35	210	196	
5	6	4	2		21	224	490	
9	6	4	2		21	224	490	
10	5	3	1		35	210	196	
11	4	2			35	112		
12	3	1			21	28		
13	2				7			
14	1				1			

## Results and discussion

**Fig. 1:**
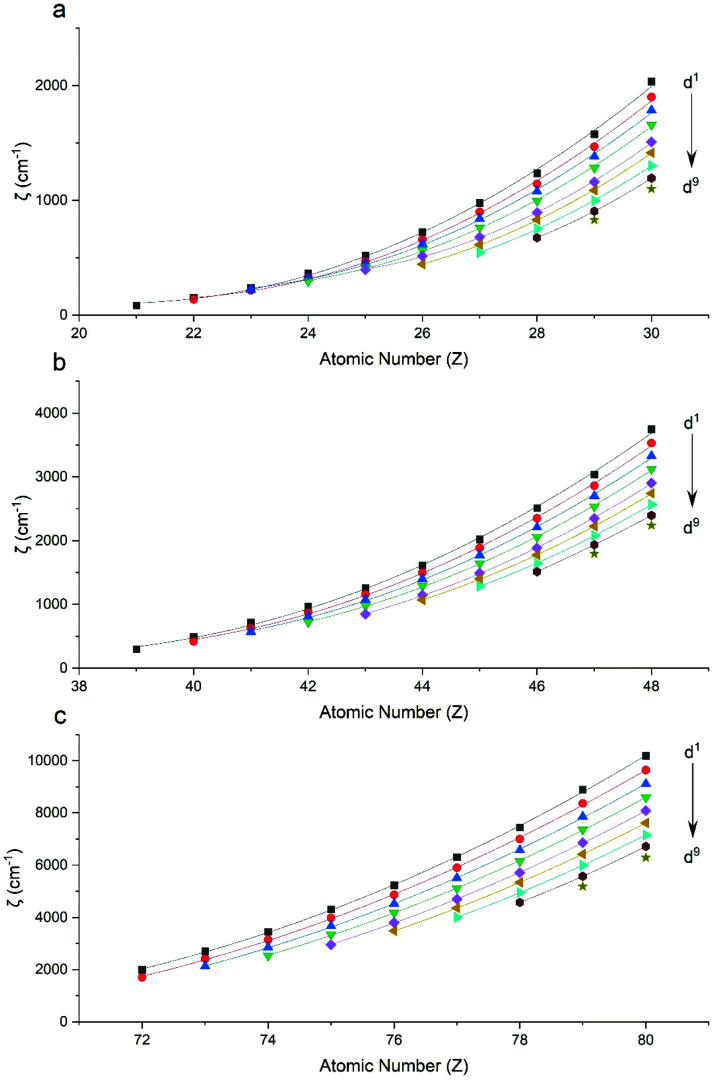
CASSCF-SO-calculated SO coupling parameters (points) for free ions (a) 3d, (b) 4d, (c) 5d. Lines are second-order polynomial fits, with parameters given in Table S5.†

Performing CASSCF-SO calculations accounting for all spin states (see Method) for a range of 3d, 4d and 4f ions and oxidations states, and mapping onto a free-ion Slater–Condon Hamiltonian (eqn (2)), we obtain the SO coupling and Slater–Condon *F*^*k*^ parameters (Tables S1–S4†). Note that the single-electron SO coupling parameter can be related to the multi-electron SO coupling parameter *via*eqn (7).^34^ We find smooth variation in the SO coupling parameters, *ζ*, as a function of atomic number, *Z* (Fig. 1 and 4), which can be well-described as a quadratic function in *Z* (Tables S5 and S7†); similar trends have been seen previously.^35,36^7
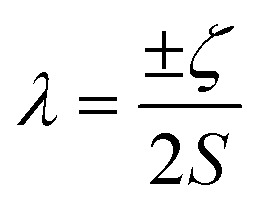


**Fig. 2:**
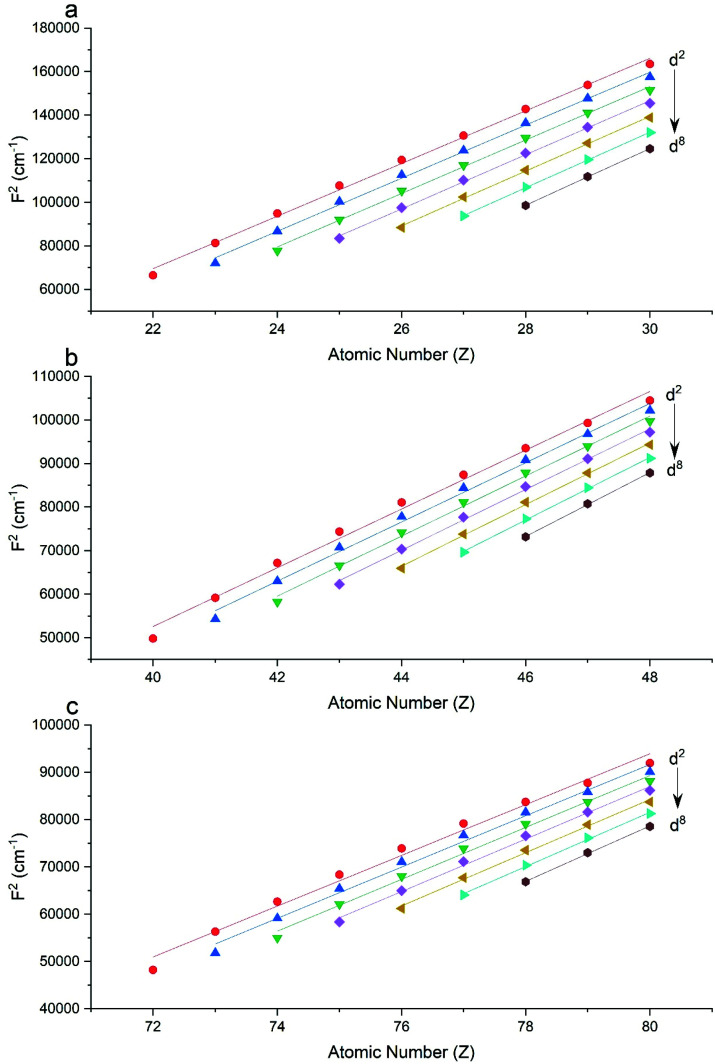
CASSCF-SO-calculated *F*^2^ parameters (points) for free ions (a) 3d, (b) 4d, (c) 5d. Lines are second-order polynomial fits, with parameters given in Table S6.†

**Fig. 3:**
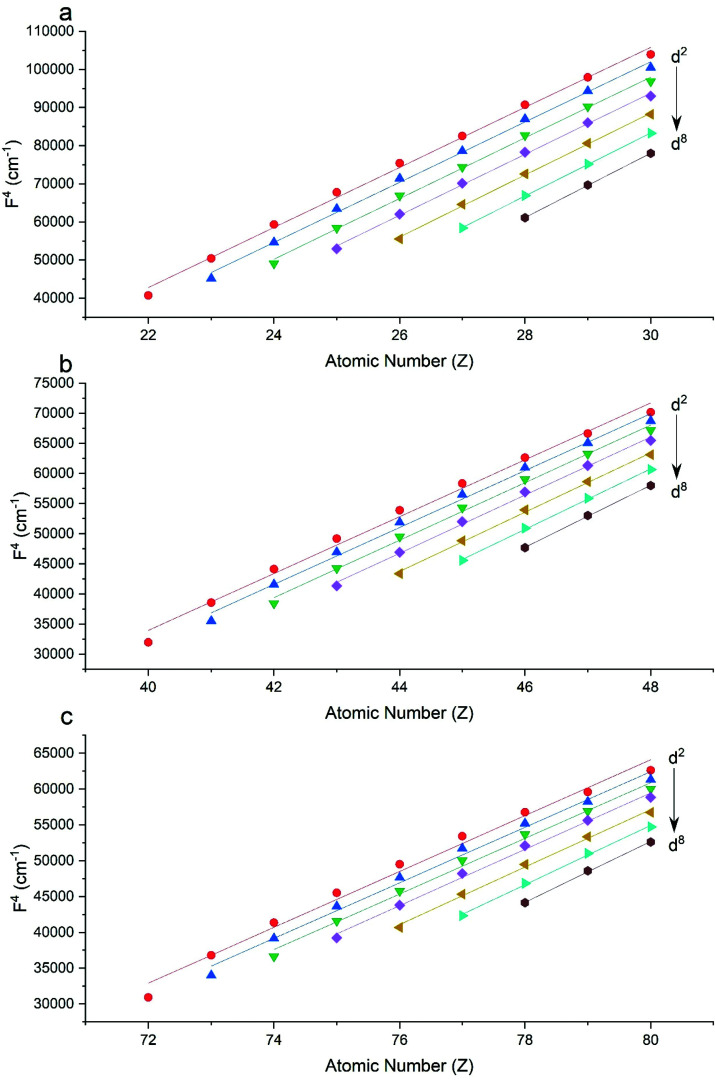
CASSCF-SO-calculated *F*^4^ parameters (points) for free ions (a) 3d, (b) 4d, (c) 5d. Lines are second-order polynomial fits, with parameters given in Table S6.†

The Slater–Condon parameters, on the other hand, show linear variation with *Z* for isoelectronic series (Fig. 2, 3 and Tables S6, S7†), a feature that has been observed before;^37^ however, there is a slight deviation from linearity that is most noticeable for the d^2^ configurations. We also find a linear trend in the Slater–Condon parameters for the trivalent 4f series where the configuration also changes as a function of *Z*(Fig. 5).

**Fig. 4:**
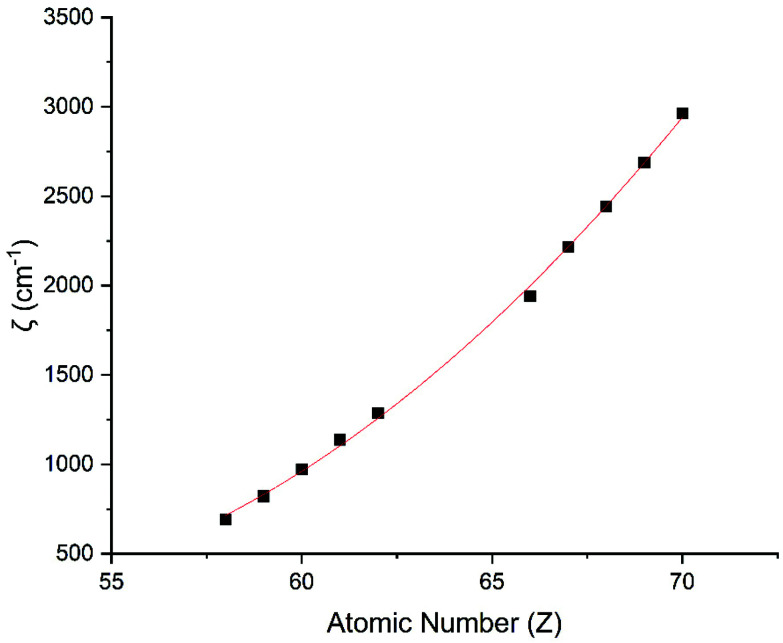
CASSCF-SO-calculated SO coupling parameters (points) for trivalent lanthanide ions. Note here that the configuration 4f^*n*^ is changing with *Z* as all ions are trivalent. Line is best fit with parameters in Table S7.†

**Fig. 5:**
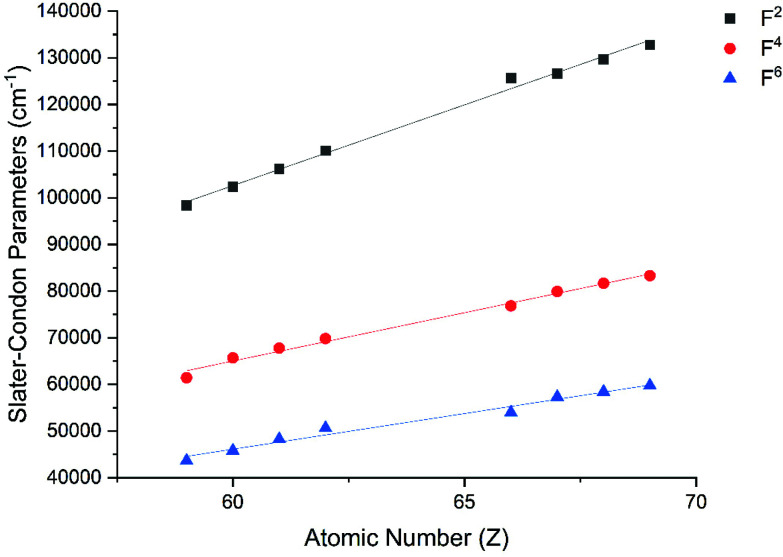
CASSCF-SO-calculated Slater–Condon (*F*^2^, *F*^4^, *F*^6^) parameters (points) for trivalent lanthanide ions. Note here that the configuration 4f^*n*^ is changing with *Z* as all ions are trivalent. Lines are best fits with parameters in Table S7.†

Compared to experiment, we find that SO coupling parameters for 3d ions are generally estimated with an error of *ca.* −20 to 60 cm^−1^ (Table S1†), with absolute relative errors on the order of 5–30% (Fig. S1†). For 4d ions, the SO coupling parameters are usually accurate within 100 cm^−1^ (with an outlier of *ca.* −200 cm^−1^ for Tc(vi)), corresponding to absolute relative errors within *ca.* 10% (Fig. S1†). The errors tend to be lower for 3d ions nearer empty or full d-shell configurations, which is likely due to averaging over fewer states in CASSCF compared to nearer half-filled configurations where there are more excited states. However, the same trend is not as clear for 4d ions, most probably because these effects are hidden in the larger absolute errors for these heavier elements. For 5d ions, the SO parameters are accurately calculated within about 200 cm^−1^ (Table S1†) which corresponds to relative errors within 10% (Fig. S1†). For trivalent 4f ions, the calculated SO coupling parameters are always overestimated by *ca.* 20–150 cm^−1^ (Table S1†) and the absolute relative errors are on the order of 1–10% (Fig. S2†), except for an outlier of Ce(iii) which is overestimated by around 20%. From these data, we can conclude that d^*n*^ and f^*n*^ SO coupling parameters are predicted to within *ca.* 200 cm^−1^ by the minimal CASSCF-SO method, which corresponds to a relatively low percentage error, usually <10% for 4d^*n*^, 5d^*n*^ and 4f^*n*^ configurations owing to the larger SO constants in the heavier elements, compared to the larger percentage error up to 30% in 3d^*n*^ configurations.

Examination of the Slater–Condon parameters show that these are subject to larger errors compared to experiment than the SO coupling parameters (Tables S2 and S3†); this is because these parameters are hugely sensitive to electron correlation, while the SO parameters are less so. Considering 3d ions, most cases, excepting a handful of *F*^4^ parameters and the *F*^2^ parameter for Co(vii), show that minimal CASSCF-SO calculations overestimate both *F*^2^ and *F*^4^ by *ca.* 2000–20 000 cm^−1^, which amounts to around 10–40% relative error in *F*^2^ (Fig. S3a†). For *F*^4^ the relative errors are larger for the underestimated values which generally occur for d^2^ configurations, whereas most values are overestimated around 5–45%. For 4d ions, for which we only have data for the divalent series, we find all the Slater–Condon parameters are overestimated, and that there appears to be a systematic error owing to the smooth trend in error (Fig. S3†). For the trivalent 4f ions, all *F*^2^, *F*^4^ and *F*^6^ parameters are overestimated by *ca.* 6000–30 000 cm^−1^, which corresponds to around 25–30% error for *F*^2^ (Table S2†), 15–20% error for *F*^4^ (Table S3†) and 12–26% error for *F*^6^ (Table S4†). We see a similar decrease in error in the Slater–Condon parameters for the trivalent 4f ions as observed for the divalent 4d ions, although there is more scatter in the 4f data (Fig. S3†), again suggesting that there is a systematic error in the estimation of the interelectronic repulsion in minimal CASSCF-SO calculations.

Given the relatively large errors in calculated Slater–Condon parameters we can hardly recommend the use of minimal active space CASSCF-SO calculations for modelling spectroscopic data. However, as the errors appear systematic for both Slater–Condon and SO parameters for trivalent 4f ions and the Slater–Condon parameters for divalent 4d, if minimal CASSCF-SO calculations are the only viable option due to computational limitations, it appears that an appropriate scaling factor on the order of 0.7–0.9 (Tables S8 and S9†) may lead to useful results, which indeed is a common approach in some communities.^38,39^ However, we do not recommend such an approach for complexes of 3d ions given that the data show rather large variations in relative error. In all cases, the errors in the calculation of the Slater–Condon parameters are due to the poor treatment of dynamic correlation in CASSCF, and as such the results can be improved if dynamic correlation can be approximated. While the accurate calculation of ionic Slater–Condon parameters is not the goal of this work, and hence we do not perform a systematic study of dynamic correlation methods here, we have performed multistate CASPT2 (MS-CASPT2)^31^ and extended multistate CASPT2 (XMS-CASPT2)^32,33^ calculations for 3d^7^ Co(ii) and 4f^2^ Pr(iii) to illustrate how the addition of corrections for dynamic correlation affects the resulting parameters (Table 4). Both MS-CASPT2 and XMS-CASPT2 are perturbative treatments to include dynamic correlation effects, however, they differ in how they are applied. MS-CASPT2 employs a state-specific Fock operator in generating the zeroth-order Hamiltonian, while XMS-CASPT2 uses a state-average Fock operator to render the zeroth-order states invariant to unitary transformations, making the XMS approach more robust in the vicinity of low-lying excitations at the cost of accuracy in cases where states are well separated; an excellent overview can be found in the discussion of a hybrid method, extended dynamically-weighted CASPT2 (XDW-CASPT2), of Battaglia and Lindh.^40^

**Table 4:** Comparison of Slater–Condon and SO parameters calculated with CASSCF, MS-CASPT2 and XMS-CASPT2 to experimental results

Parameter	Experiment (cm^−1^)	CASSCF (cm^−1^)	MS-CASPT2 (cm^−1^)	XMS-CASPT2 (cm^−1^)	AILFT-NEVPT2^42^ (cm^−1^)
**Co(ii)**					
*F* ^2^	79 037	93 743	79 400	79 602	—
*F* ^4^	56 889	58 407	52 227	53 543	—
*ζ*	515	545	662	771	—
**Pr(iii)**					
*F* ^2^	68 323	98 391	82 429	84 486	72 980
*F* ^4^	49 979	61 439	47 971	51 707	56 740
*F* ^6^	32 589	43 365	33 181	35 686	38 126
*ζ*	747	822	823	883	800

The results for Pr(iii) show that the electronic-repulsion is much more accurately modelled when dynamic correlation is accounted for, reducing the absolute error in *F*^2^, *F*^4^ and *F*^6^ from 44, 23 and 33%, respectively, for CASSCF, to 21, 4 and 2% for MS-CASPT2 and 24, 3 and 10% for XMS-CASPT2. However, both CASPT2 variants fail to maintain the degeneracy of SO eigenstates (each ^2*S*+1^*L*_*J*_ term should be (2*J* + 1)-fold degenerate in spherical symmetry), and hence this can have a detrimental effect on the prediction of the SO coupling parameter; the 10% error at the CASSCF level is maintained at 10% for MS-CASPT2, but increases to 18% for XMS-CASPT2. There are similar improvements for Co(ii): the absolute error in *F*^2^ and *F*^4^ changes from 19 and 3%, respectively, for CASSCF, to <1% and 8% for MS-CASPT2 and <1% and 6% for XMS-CASPT2. However, in this case the error in the prediction of the SO parameter increases from 6% at the CASSCF level to 29% and 50% for MS-CASPT2 and XMS-CASPT2, respectively. It is expected that CASPT2 variants will be more reliable in molecular systems where the CF removes the high degeneracy of ionic electronic terms, and thus perturbation methods are more reliable, however extreme care must be taken in the case of near-degenerate states.

We now compare our results to those obtained using the related methods of AILFT and LF-DFT. AILFT as developed by Atanasov, Ganyushin, Sivalingam and Neese,^17^ is a module of the Orca program that is able to re-cast a minimal active space CASSCF calculation into a ligand field (a.k.a. crystal field) model Hamiltonian. This method partitions the configuration state functions (CSF) of a CASSCF calculation into those arising from a d^*n*^ or f^*n*^ configuration, and the remaining ones (the “outer space”), and then approximates an effective Hamiltonian in the d^*n*^ or f^*n*^ CSF space which can be mapped in a one-to-one fashion with a model Hamiltonian (such as eqn (2)) in order to extract the Slater–Condon and spin–orbit coupling parameters (and crystal or ligand field parameters). The main difference between AILFT and the present method is that AILFT approximates the effective *ab initio* Hamiltonian in the restricted CSF space before projecting onto a model Hamiltonian, while the present method directly projects the *ab initio* states onto a model Hamiltonian, and thus it is in the alignment between the *ab initio* and the model states where the approximation is made in the present case. Each approach has its advantages and disadvantages: while the present method makes no approximations in the construction of the effective Hamiltonian (subject to the premise that the *ab initio* Hamiltonian can be approximated by a model Hamiltonian at all), it is not as simple to perform the projection when the *ab initio* states are polluted by states outside the proposed model space. AILFT has no such problems as the model space is always well defined, however it requires approximation of the effective Hamiltonian. Thus, despite both AILFT and the present method deriving from CASSCF wavefunctions, they take rather different approaches to obtain the model parameters. None-the-less, the two methods provide very similar results: the AILFT-calculated *F*^2^ and *F*^4^ parameters for Cr(iii), Mo(iii) and W(iii) are within 1% of those from the present method,^41^ and the *F*^2^, *F*^4^ and *F*^6^ parameters for the series of trivalent 4f ions agree within 4% to the present method (average discrepancy is <1%).^42^ We have shown that our minimal CASSCF-SO calculations significantly overestimate the interelectronic repulsion and hence the Slater–Condon parameters, which can be improved by perturbative corrections for dynamic correlation, and the AILFT method is no different; addition of NEVPT2 corrections to the reference wavefunction improves the predicted parameters for the Pr(iii) free ion (Table 4), where the calculated value of *F*^2^ is the closest of all methods to the experimental one, however the present calculations with MS-CASPT2 corrections appear to have an edge over NEVPT2 when it comes to the *F*^4^ and *F*^6^ parameters. We have found that the SO parameters obtained herein (which employs the AMFI method^30^ to obtain the SO Hamiltonian) are accurate to within *ca.* 10% of the experimental data, while it is reported that the SO parameters obtained from AILFT using the SO mean-field (SOMF) method are accurate to within 5% of experiment;^42^ this suggests that the latter method is more accurate for SO coupling in the 4f ions.

The second method LF-DFT, developed by Atanasov, Daul and Rauzy,^43,44^ is rather different to AILFT and the method used herein because it is based on DFT calculations. Simply, the LF-DFT method determines a set of d- or f-based Kohn–Sham orbitals using a spin-restricted average-of-configuration calculation (where each d or f orbital carries *n*/5 or *n*/7, respectively, electrons), and subsequently calculates the energies of all possible Slater determinants in these d or f orbitals using a spin-unrestricted formalism.^43,44^ From these energies, the Slater–Condon and SO coupling^45^ parameters (and crystal or ligand field parameters) can be extracted. Most comparisons of this method have been made on complexes of metal ions rather than free ions, however one of the early papers presents data for Cr(iv), Mn(v) and Fe(vi) using the LDA density-functional. The LF-DFT(LDA)-calculated Slater–Condon parameters are very good for Cr(iv), coming within 3% for both *F*^2^ and *F*^4^ compared to experiment, however the method underestimates the parameters for Mn(v) and Fe(vi) by 10–20%. In contrast, the calculations herein overestimate *F*^2^ and *F*^4^ by 10–20% for Cr(iv), while overestimating *F*^2^ and underestimating *F*^4^ by around 10% and 20%, respectively, for both Mn(v) and Fe(vi). Thus, overall, the results of LF-DFT(LDA) are of a similar quality to those obtained with minimal CASSCF-SO, with the caveat that LF-DFT will be subject to a user choice of density-functional which does not offer systematic improvement, unlike the ability to add dynamic correlation with CASSCF methods *via* perturbation theory. However, it should be noted that in real applications LF-DFT will almost certainly always outperform CASSCF methods in efficiency. Indeed, the LF-DFT method has recently been shown to outperform the accuracy of commonplace time-dependent DFT (TD-DFT) calculations for d–d optical spectra, and approaches the accuracy of more advanced multiconfigurational methods.^46^

## Conclusions

Herein we have performed minimal active space CASSCF-SO calculations with OpenMolcas to determine Slater–Condon and SO coupling parameters for metal ions from across the periodic table. We find that the variance of SO coupling parameters as a function of atomic number for a given d^*n*^ configuration is well-described by a quadratic function, while the Slater–Condon parameters show a linear variation. Comparison to experimentally-derived parameters show that the SO coupling is predicted to within *ca.* 200 cm^−1^ by the minimal CASSCF-SO method, corresponding usually to <10% error for 4d^*n*^, 5d^*n*^ and 4f^*n*^ configurations, but the relative error is larger in the case of 3d^*n*^ configurations, up to 30%, owing to the smaller SO coupling. On the other hand, Slater–Condon parameters are usually overestimated with larger relative errors on the order of 10–50%. These significant errors arise due to the well-known lack of dynamic correlation in CASSCF calculations, and as such we cannot recommend minimal active space CASSCF-SO for applications where high-energy spectroscopic data are concerned. However, there are systematic errors in the calculated Slater–Condon for divalent 4d and trivalent 4f ions, such that scaling may lead to useful results in cases where minimal CASSCF-SO calculations are the only viable option. We show that (X)MS-CASPT2 corrections can substantially improve the calculated Slater–Condon parameters, but that the calculated SO parameters are detrimentally affected, and observe significant symmetry breaking of the SO manifolds, so care should be taken using such methods in cases of high symmetry.

## Conflicts of interest

There are no conflicts to declare.

## Supplementary Material

DT-050-D1DT02346B-s001
