# Supplementary material for: Assessment of minimal active space CASSCF-SO methods for calculation of atomic Slater–Condon and spin–orbit coupling parameters in d- and f-block ions
Source: Dalton Trans. 2021 Oct 1;50(40):14130–8. doi: 10.1039/d1dt02346b (PMC9583075; doi:10.1039/d1dt02346b)
Supplement: DT-050-D1DT02346B-s001 [file DT-050-D1DT02346B-s001.pdf]

Electronic Supplementary Material (ESI) for Dalton Transactions.
This journal is © The Royal Society of Chemistry 2021

Supporting Information for:

Assessment of minimal active space CASSCF-SO methods for calculation of atomic Slater-Condon and spin-orbit coupling parameters in d- and f-block ions

Alvin J. Walisinghe[a] and Nicholas F. Chilton[a,*]

[a] Department of Chemistry, School of Natural Sciences, The University of Manchester, Oxford Road, Manchester, M13 9PL, UK

email: nicholas.chilton@manchester.ac.uk

Table S1. Comparison of calculated and experimentally-derived SO coupling parameters.

Ion		Ref	Experimental (cm^{-1})						Calculated (cm^{-1})										Difference (cm^{-1})					
			II	III	IV	V	VI	VII	II	III	IV	V	VI	VII	VIII	IX	X	XI	II	III	IV	V	VI	VII
3d	Sc	1	80						83										3					
3d	Ti	1	120	155					136	152									16	-3				
3d	V	1	170	210	250				221	215	238								51	5	-12			
3d	Cr	1	230	275	325	380			293	323	330	364							63	48	5	-16		
3d	Mn	1		355	415	475	540		396	415	448	470	520							60	33	-5	-20	
3d	Fe	1, 2	400		515	555	665	734	443	515	565	617	658	723					43		50	62	-7	-11
3d	Co	1	515			715	790		545	613	679	763	840	899	978				30			48	50	
3d	Ni	1	630				950		675	753	831	894	997	1077	1143	1235			45				47	
3d	Cu	1	830						829	905	996	1087	1160	1283	1381	1466	1576		-1					
3d	Zn	3		1104						1099	1193	1302	1414	1508	1658	1786	1900	2035		-5				
4d	Y	1	300						296										-4					
4d	Zr	1	425	500					422	489									-3	-11				
4d	Nb	1	555	670	750				570	633	715								15	-37	-35			
4d	Mo	1		820	950	1030			721	809	880	967								-11	-70	-63		
4d	Tc	1					1450		850	979	1075	1159	1257										-193	
4d	Ru	1	1000						1070	1149	1289	1400	1500	1615					70					
4d	Rh	1	1220						1287	1404	1493	1645	1771	1888	2020				67					
4d	Pd	1	1460						1515	1645	1774	1887	2057	2208	2353	2513			55					
4d	Ag	1	1840						1797	1937	2083	2226	2350	2563	2701	2861	3036		-43					
4d	Cd	1		2325						2240	2397	2567	2742	2906	3124	3329	3532	3747		-85				

Table S1. Cont.

Ion		Ref.	Experimental (cm^{-1})						Calculated (cm^{-1})										Difference (cm^{-1})					
			II	III	IV	V	VI	VII	II	III	IV	V	VI	VII	VIII	IX	X	XI	II	III	IV	V	VI	VII
5d	Hf								1703	1996														
	Ta								2127	2419	2711													
	W	4–6	2364	2720	3102				2537	2852	3142	3451							173	132	40			
	Re	7–9	2839	3210	3593				2963	3344	3672	3983	4313						124	134	79			
	Os	10	3343						3484	3805	4178	4524	4865	5228					141					
	Ir	11		4307					4003	4363	4706	5121	5513	5903	6314					56				
	Pt	12–16		4926	5354	5797	6242	6701	4575	4956	5341	5715	6157	6580	7004	7447				30	-13	-82	-85	-121
	Au								5183	5579	5992	6425	6860	7362	7859	8364	8890							
	Hg	17		6274						6293	6720	7160	7621	8083	8600	9117	9644	10190		19				
4f	Ce	18		644						691										147				
	Pr	19		747						822										75				
	Nd	19		879						971										92				
	Pm	19		1023						1138										115				
	Sm	19		1170						1287										117				
	Dy	19		1915						1940										25				
	Ho	19		2142						2215										73				
	Er	19		2358						2443										85				
	Tm	19		2644						2689										45				
	Yb	18		2918						2963										45				

Table S2. Comparison of calculated and experimentally-derived F^2 Slater-Condon parameters.

Ion		Ref.	Experimental (cm^{-1})							Calculated (cm^{-1})									Difference (cm^{-1})						
			II	III	IV	V	VI	VII	VIII	II	III	IV	V	VI	VII	VIII	IX	X	II	III	IV	V	VI	VII	VIII
3d	Sc																								
	Ti	[20]	49784							66442									16658						
	V	[20, 21]	59829	64925						72124	81266								12295	16341					
	Cr	[20, 21]	64631	73892	79576					77747	86741	94887							13116	12849	15311				
	Mn	[22, 20, 21]	69090	78449	94815	101038				83370	92084	100296	107691						14280	13635	5481	6653			
	Fe	[22, 21]	73206	83349			117698			88418	97524	105316	112629	119464					15212	14175			1766		
	Co	[22, 20, 21]	79037	88004		94374	109760	130830		93743	102454	110260	117044	123837	130611				14706	14450		22670	14077	-219	
	Ni	[22, 20, 21]	84427	92806			107555		140679	98591	106941	114804	122590	129603	136447	142841			14164	14135			22048		2162
	Cu										111827	119630	127120	134521	141190	147722	153876								
	Zn											124609	132013	138910	145544	151543	157588	163501							
4d	Y																								
	Zr	[23]	34790							49826									15036						
	Nb	[23]	39935							54287	59156								14352						
	Mo	[23]	45080							58218	63001	67161							13138						
	Tc	[23]	50225							62270	66628	70710	74370						12045						
	Ru	[23]	55370							65934	70365	74177	77797	81067					10564						
	Rh	[23]	60515							69640	73797	77686	81117	84424	87445				9125						
	Pd	[23]	65660							73162	77299	81111	84725	87889	90856	93527			7502						
	Ag										80770	84434	87831	91100	93987	96770	99323								
	Cd											87857	91216	94293	97198	99743	102195	104471							

Table S2. Cont.

Ion		Ref.	Experimental (cm^{-1})							Calculated (cm^{-1})									Difference (cm^{-1})						
			II	III	IV	V	VI	VII	VIII	II	III	IV	V	VI	VII	VIII	IX	X	II	III	IV	V	VI	VII	VIII
5d	Hf									48233															
	Ta									51818	56302														
	W									55019	59145	62663													
	Re									58348	62118	65428	68369												
	Os									61205	64994	68055	71072	73889											
	Ir									64058	67705	71110	73920	76685	79156										
	Pt									66848	70348	73528	76583	79087	81545	83751									
	Au										73016	76114	78920	81588	83731	85828	87742								
	Hg											78560	81269	83738	86149	88136	90103	91941							
4f	Pr	[19]		68323							98391									30068					
	Nd	[19]		72295							102327									30032					
	Pm	[19]		75842							106206									30364					
	Sm	[19]		79012							110078									31066					
	Dy	[19]		92373							125614									33241					
	Ho	[19]		95772							126625									30853					
	Er	[19]		97909							129710									31801					
	Tm	[19]		101381							132784									31403					

Table S3. Comparison of calculated and experimentally-derived F^4 Slater-Condon parameters.

Ion		Ref	Experimental (cm^{-1})							Calculated (cm^{-1})									Difference (cm^{-1})						
			II	III	IV	V	VI	VII	VIII	II	III	IV	V	VI	VII	VIII	IX	X	II	III	IV	V	VI	VII	VIII
	Sc																								
	Ti	[22]	36603							40728									4125						
	V	[22, 21]	41013	52479						45144	50429								4131	-2050					
	Cr	[20, 21]	44982	52038	55566					49130	54608	59331							4148	2570	3765				
	Mn	[22, 20, 21]	48510	56007	48069	79821				52954	58393	63439	67755						4444	2386	15370	-12066			
3d	Fe	[22, 21]	50715	60417			93051			55509	62075	66928	71406	75402					4794	1658			-17649		
	Co	[22, 21]	56889	64386				103194		58407	64586	70137	74395	78572	82545				1518	200				-20649	
	Ni	[22, 21]	61299	68796					107604	61080	66920	72593	78293	82759	86973	90707			-219	-1876					-16897
	Cu										69676	75180	80611	86043	90285	94300	97888		4125						
	Zn											78001	83234	88197	93001	96837	100478	103916	4131	-2050					
	Y																								
	Zr	[23]	23373							31945									8572						
	Nb	[23]	26901							35505	38539								8604						
	Mo	[23]	30429							38427	41567	44137							7998						
	Tc	[23]	33957							41324	44241	46928	49198						7367						
4d	Ru	[23]	37485							43370	46920	49489	51872	53897					5885						
	Rh	[23]	41013							45552	48830	51997	54315	56491	58356				4539						
	Pd	[23]	44541							47679	50911	53960	56941	59042	60972	62629			3138						
	Ag										53015	55892	58620	61320	63250	65058	66635								
	Cd											57991	60635	63106	65476	67170	68731	70157							

Table S3. Cont.

Ion		Ref.	Experimental (cm^{-1})							Calculated (cm^{-1})									Difference (cm^{-1})						
			II	III	IV	V	VI	VII	VIII	II	III	IV	V	VI	VII	VIII	IX	X	II	III	IV	V	VI	VII	VIII
5d	Hf									30926															
	Ta									33996	36801														
	W									36651	39153	41375													
	Re									39224	41610	43616	45514												
	Os									40695	43781	45785	47647	49511											
	Ir									42326	45327	48206	50043	51711	53431										
	Pt									44132	46851	49494	52104	53692	55210	56763									
	Au										48603	51017	53340	55622	56926	58232	59608								
	Hg											52622	54726	56744	58837	60038	61319	62626							
4f	Pr	19		49979							61439									11460					
	Nd	19		52281							65659									13378					
	Pm	19		54319							67772									13453					
	Sm	19		56979							69811									12832					
	Dy	19		59401							76815									11534					
	Ho	19		60517							79917									12405					
	Er	19		63485							81664									11315					
	Tm	19		65281							83320									13090					

Table S4. Comparison of calculated and experimentally-derived F^6 Slater-Condon parameters for trivalent 4f ions.

Ion (3+)	Ref.	Experimental (cm^{-1})	Calculated (cm^{-1})	Difference (cm^{-1})
**Pr**	[19]	32589	43365	11056
**Nd**	[19]	35374	45804	10430
**Pm**	[19]	38945	48262	9317
**Sm**	[19]	40078	50638	10560
**Dy**	[19]	47642	53981	6339
**Ho**	[19]	48582	57312	8730
**Er**	[19]	48861	58431	9570
**Tm**	[19]	51827	59748	7921

Table S5. Fitted quadratic parameters for CASSCF-SO-calculated SO coupling parameters, for an equation of the form $\zeta = aZ^2 + bZ + c$.

Group	Configuration (d^n)	a	b	c
3d	1	21.56	-890	9293
	2	22.25	-943	10119
	3	23.17	-1010	11197
	4	24.01	-1073	12223
	5	27.73	-1305	15692
	6	25.14	-1166	13775
	7	24.50	-1145	13604
	8	29.00	-1423	17783
4d	1	28.91	-2143	39937
	2	29.77	-2240	42408
	3	30.35	-2314	44436
	4	31.04	-2397	44436
	5	31.45	-2454	48223
	6	31.86	-2514	50026
	7	31.50	-2502	50074
	8	19.00	-1345	23181
5d	1	54.13	-7205	340230
	2	50.51	-6689	221567
	3	50.46	-6725	224138
	4	49.95	-6685	223735
	5	51.45	-6952	234992
	6	52.86	-7212	246303
	7	53.75	-7388	254202
	8	68.50	-9751	348360

Table S6. Fitted linear parameters for CASSCF-SO calculated Slater-Condon parameters, for an equation of the form $F^k = aZ + b$.

	Configuration (d^n)	F^2		F^4	
Group	n	a	b	a	b
3d	2	12082	-196279	7878	-130524
	3	12176	-205500	7900	-134974
	4	12282	-215244	7955	-140677
	5	12405	-225515	8008	-146481
	6	12565	-237479	8140	-155624
	7	12750	-250290	8274	-164877
	8	13009	-365585	8461	-175769
4d	2	6748	-217324	4721	-154905
	3	6801	-222630	4724	-156813
	4	6893	-229935	4779	-161331
	5	6968	-236492	4826	-165579
	6	7075	-244866	4926	-173028
	7	7186	-253516	5023	-180322
	8	7348	-264736	5156	-189437
5d	2	5369	-335563	3899	-247785
	3	5421	-342013	3875	-247579
	4	5486	-349558	3882	-249676
	5	5550	-357018	3928	-254804
	6	5628	-365973	4011	-263746
	7	5740	-377635	4164	-275993
	8	5865	-389816	4245	-286903

Table S7. Fitted linear and quadratic parameters for CASSCF-SO calculated Slater-Condon parameters (for an equation of the form $F^k = aZ + b$) and SO coupling parameters (for an equation of the form $\zeta = aZ^2 + bZ + c$) in Ln^{3+} ions.

F^2		F^4		F^6	
a	b	a	b	a	b
3461	-105060	2073	-59363	1526	-45467
SO coupling constant (ζ)					
a		b		c	
6.24		-614		15302	

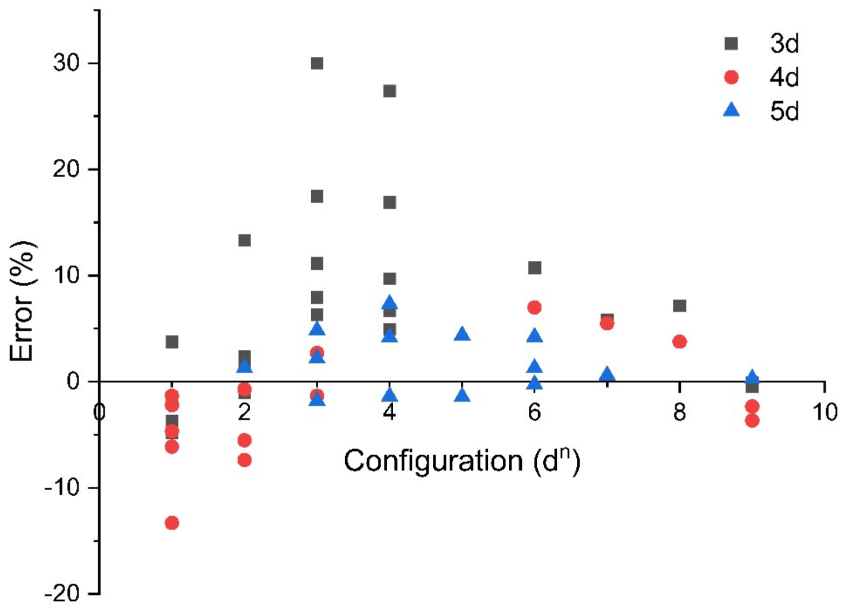

Figure S1. Percentage error in calculated SO parameters for 3d, 4d and 5d ions.

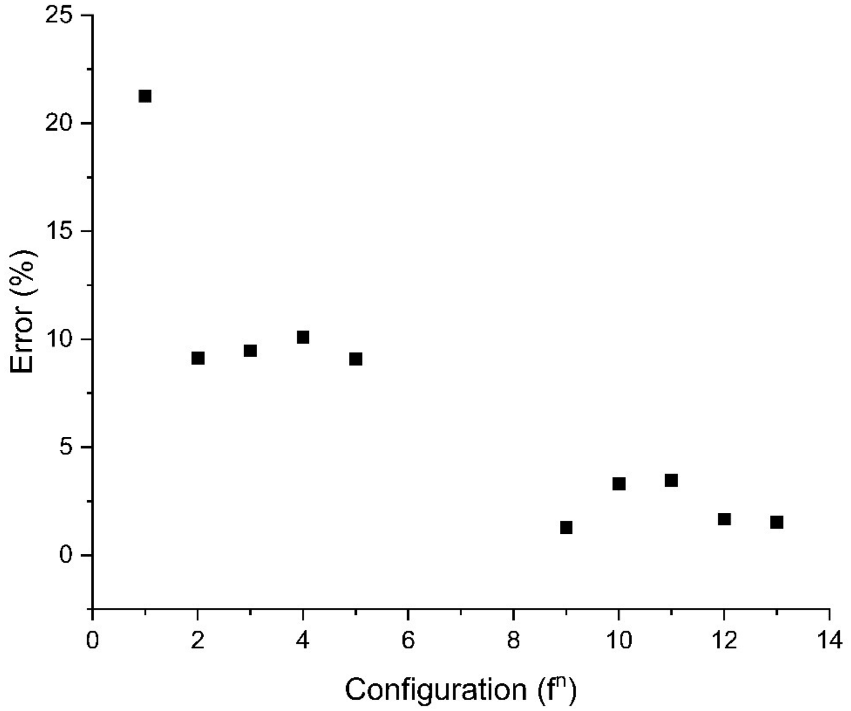

Figure S2. Percentage error in calculated SO parameters for Ln^{3+} ions.

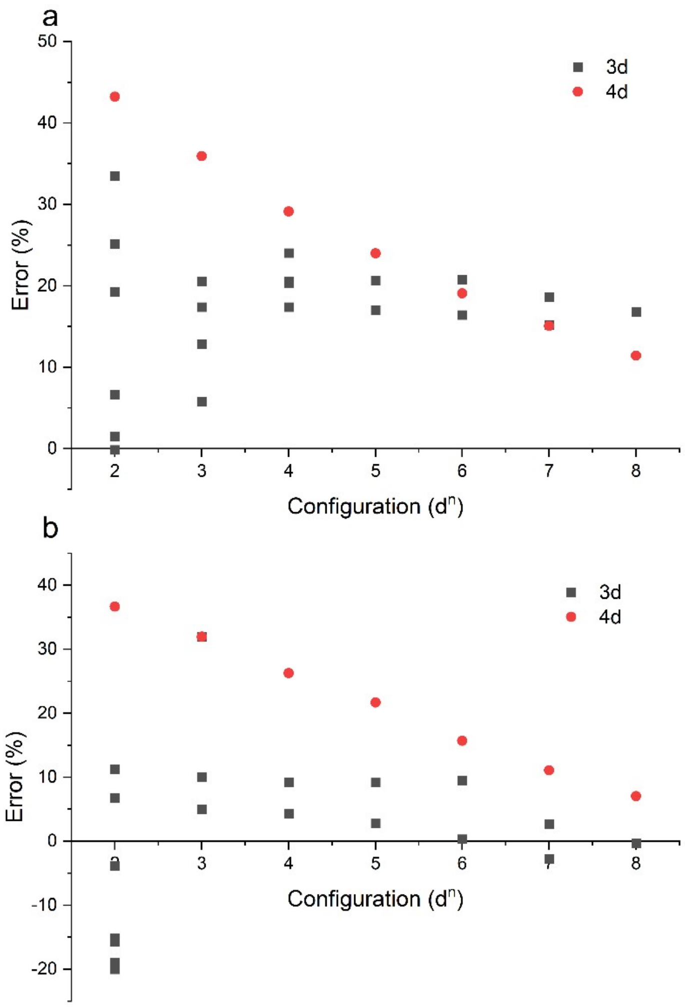

Figure S3. Percentage error in calculated Slater-Condon parameters (a) F^2 and (b) F^4.

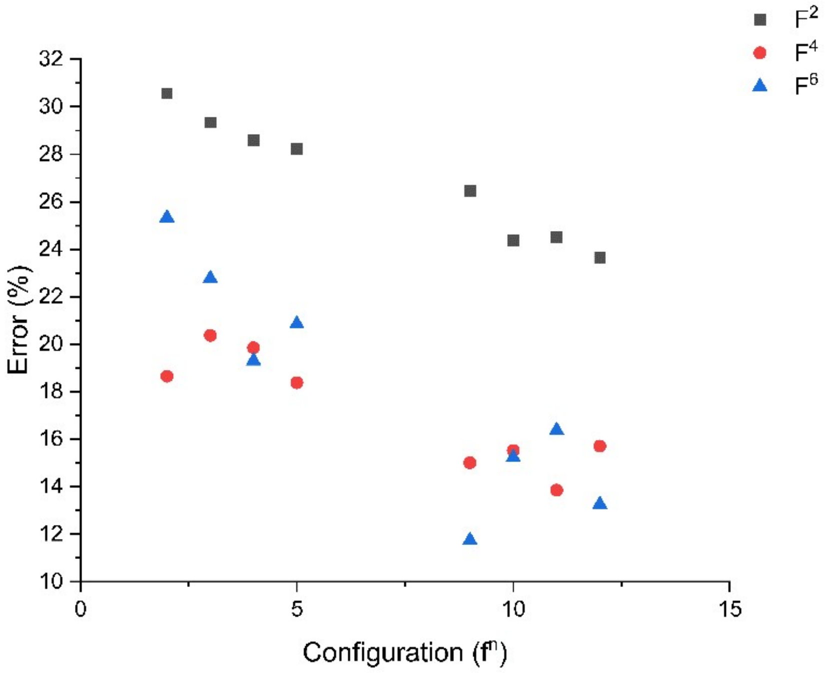

Figure S4. Percentage error in calculated Slater-Condon parameters for Ln^{3+} ions.

Table S8. Suggested scaling factors to apply to minimal CASSCF-SO-calculated Slater-Condon parameters for divalent 4d ions.

**Ion (2+)**	F^2 and F^4
Zr	0.71
Nb	0.75
Mo	0.78
Tc	0.81
Ru	0.85
Rh	0.88
Pd	0.92

Table S9. Suggested scaling factors to apply to minimal CASSCF-SO-calculated Slater-Condon and SO parameters for trivalent 4f ions.

**Ion (3+)**	F^2, F^4 and F^6	ζ
Ce	-	0.93
Pr	0.75	0.91
Nd	0.76	0.91
Pm	0.77	0.90
Sm	0.78	0.91
Dy	0.80	0.99
Ho	0.79	0.97
Er	0.79	0.97
Tm	0.80	0.98
Yb	-	0.98

References

1 T. M. Dunn, *Trans. Faraday Soc.*, 1961, **57**, 1441–1444.
2 J. Sugar and C. Corliss, *Atomic energy levels of the iron-period elements: potassium through nickel*, United States, 1985.
3 J. Sugar and A. Musgrove, *J. Phys. Chem. Ref. Data*, 1995, **24**, 1803–1872.
4 A. S. King and R. B. King, *Astrophys. J.*, 1932, **75**, 40.
5 L. Iglesias, V. Kaufman, O. Garcia-Riquelme and F. R. Rico, *Phys. Scr.*, 1985, **31**, 173–183.
6 F. G. Meijer, *Phys. BC*, 1986, **141**, 230–236.
7 V. I. Azarov and R. R. Gayasov, *At. Data Nucl. Data Tables*, 2018, **121–122**, 306–344.
8 V. I. Azarov and R. R. Gayasov, *At. Data Nucl. Data Tables*, 2018, **119**, 218–249.
9 V. I. Azarov and R. R. Gayasov, *At. Data Nucl. Data Tables*, 2018, **119**, 175–192.
10 V. I. Azarov, W.-Ü. L. Tchang-Brillet and R. R. Gayasov, *At. Data Nucl. Data Tables*, 2018, **121–122**, 345–377.
11 V. I. Azarov and R. R. Gayasov, *At. Data Nucl. Data Tables*, 2016, **108**, 81–117.
12 V. I. Azarov and R. R. Gayasov, *At. Data Nucl. Data Tables*, 2016, **108**, 118–153.
13 V. I. Azarov and R. R. Gayasov, *At. Data Nucl. Data Tables*, 2016, **108**, 154–192.
14 V. I. Azarov and R. R. Gayasov, *At. Data Nucl. Data Tables*, 2017, **115–116**, 309–343.
15 V. I. Azarov and R. R. Gayasov, *At. Data Nucl. Data Tables*, 2017, **115–116**, 344–368.
16 V. I. Azarov and R. R. Gayasov, *At. Data Nucl. Data Tables*, 2017, **115–116**, 369–384.
17 Y. N. Joshi, A. J. J. Raassen and B. Arcimowicz, *J. Opt. Soc. Am. B*, 1989, **6**, 534–538.
18 W. C. Martin, R. Zalubas and L. Hagan, *Natl Bur Stand Natl Stand Ref Data Ser.*
19 C. Görller-Walrand and K. B. T.-H. on the P. and C. of R. E. Binnemans, Elsevier, 1996, vol. 23, pp. 121–283.
20 M. A. Catalán and M. T. Antunes, *Z. Für Phys.*, 1936, **102**, 432–460.
21 Y. Tanabe and H. Kamimura, *J. Phys. Soc. Jpn.*, 1958, 13, 539.
22 W. M. Cady, *Phys. Rev.*, 1933, **43**, 322–328.
23 E. Tondello, G. De Michelis, L. Oleari and L. Di Sipio, *Coord. Chem. Rev.*, 1967, **2**, 53–63.
